# P-1084. Long Story Short: How Long Should We Treat VRE Bacteremia?

**DOI:** 10.1093/ofid/ofaf695.1279

**Published:** 2026-01-11

**Authors:** Eleazar Torres, M Gabriela Cabanilla, Nicole Hlavacek

**Affiliations:** University of New Mexico Hospital, Albuquerque, NewMexico; University of New Mexico Health Sciences Center, Albuquerque, NM; University of New Mexico Hospital, Albuquerque, NewMexico

## Abstract

**Background:**

The optimal duration of antimicrobial therapy for uncomplicated vancomycin-resistant Enterococcus (VRE) bacteremia remains uncertain, as existing guidelines offer limited direction for managing nosocomial bloodstream infections. This study compared the clinical outcomes of short-course (≤ 6 days) and long-course ( > 6 days) antibiotic therapy for uncomplicated VRE bacteremia.

**Methods:**

We conducted a retrospective study of adult hospitalized patients with uncomplicated VRE bacteremia at the University of New Mexico Hospital between January 2014 and August 2024. The primary outcome was bacteremia recurrence within 30 days of treatment completion. Secondary outcomes included infection-related hospital readmission within 30 days, 30-day all-cause mortality, post-bacteremia hospital length of stay (LOS), and antibiotic-associated adverse events. Statistical analyses included descriptive statistics, chi-squared tests, and Fisher’s exact tests.

**Results:**

A total of 48 patients met the inclusion criteria, with 10 patients receiving short-course therapy (≤ 6 days) and 38 receiving long-course therapy ( > 6 days). The median treatment durations were 5 days (IQR 4–6) and 12 days (IQR 6–18), respectively. No recurrence of bacteremia occurred in either group. Infection-related readmission occurred in 7.9% (n = 3) of the long-course patients, but not in the short-course group (p = 0.49). Thirty-day all-cause mortality rate was 30% (n = 3) in the short-course group and 21.1% (n = 8) in the long-course group (p = 0.41). Median total hospital LOS was shorter in the short-course group (14.2 vs. 27.8 days, p = 0.48), with a similar trend in post-bacteremia LOS (10.5 vs. 13 days, p = 0.68). Clostridioides difficile infections were documented in 20% (n = 2) of short-course patients and 7.9% (n = 3) of long-course patients (p = 0.28). No cases of rhabdomyolysis or serotonin syndrome were identified in either group.

**Conclusion:**

Short-course therapy (≤ 6 days) for uncomplicated VRE bacteremia was associated with no recurrence and comparable mortality to longer durations. These findings support the need for prospective studies to validate the optimal treatment duration and inform antimicrobial stewardship efforts.

**Disclosures:**

M. Gabriela Cabanilla, PharmD, PhC, Merck & Co, Inc: Advisor/Consultant|Merck & Co, Inc: Grant/Research Support

